# Sizing Up Extracellular DNA: Instant Chromatin Discharge From Cells When Placed in Serum-Free Conditions

**DOI:** 10.3389/fcell.2020.00634

**Published:** 2020-07-22

**Authors:** Giannis Spyrou, Daniel Appelgren, Anders Rosén, Björn Ingelsson

**Affiliations:** ^1^Department of Biomedical and Clinical Sciences, Linköping University, Linköping, Sweden; ^2^Department of Health, Medicine and Caring Sciences, Linköping University, Linköping, Sweden

**Keywords:** cell death, chromatin structure, extracellular DNA, leukocytes, serum-free conditions

## Abstract

How do you wash cells? Three out of four of our colleagues use experimental procedures during everyday lab-bench work that can severely impair data interpretation depending on how cells are handled. We show here that a subpopulation (2–3%) of human leukocytes immediately induce a yet unclassified lytic cell death, concomitant with discharge of chromatin entities and cell elimination, when placed in protein-free solutions (i.e., PBS and HBSS). DNA release was not restricted to hematopoietic cells but occurred also in HEK293T cells. Albumin, fetal bovine serum, polyethylene glycol, and Pluronic F-68 supplements prevented chromatin discharge. Expelled chromatin was devoid of surrounding membranes but maintained its original nuclear shape, although ∼10 times enlarged. These structures differed from DNA appearance after osmotic or detergent-induced cell lysis. Besides sounding a cautionary note to the neutrophil extracellular trap (NET) research community, in which ∼50% of all published studies used protein-free media for NET-formation, our study also provides a rapid tool for analysis of chromatin organization.

## Introduction

Cell deaths are generally divided into two main categories: regulated cell death (RCD) and accidental cell death (ACD). While the latter represents an instant and dramatic event due to chemical, physical, or mechanical nature (i.e., osmotic and shear forces), RCD depends on highly controlled molecular machineries (i.e., apoptosis and autophagy-dependent cell death) (Galluzzi et al., 2018). While some RCD display a more apoptotic morphology, others show necrotic characteristics. One example of an RCD exhibiting necrotic features is NETotic cell death. According to the recommendations of the Nomenclature Committee on Cell Death 2018, NETotic cell death is defined as a reactive oxygen species (ROS)-dependent modality of RCD restricted to cells of hematopoietic derivation and associated with neutrophil extracellular trap (NET) extrusion (Galluzzi et al., 2018). NETs were first described as a defense mechanism utilized by neutrophils to combat infections by trapping and killing an invading pathogen (Brinkmann et al., 2004). Recently, this research-field has expanded dramatically, increasing the list of leukocytes that actively release DNA (both of nuclear and mitochondrial origin) in response to a variety of stimuli (Boeltz et al., 2019). Although there is a strong consensus among researchers within the NET community with regard to composition, morphology and triggers of NETs, there is an experimental diversity concerning culture conditions used for *in vitro* NET formation. Therefore, a call for introducing standardized buffers has been raised (Boeltz et al., 2019). Formation of NETs are mostly performed at low serum concentrations, based on early reports on concentration-dependent inhibition of NET-formation by serum, possibly due to heat-stable nucleases that degrade NETs (Fuchs et al., 2007; von Kockritz-Blickwede et al., 2009). Hence, it was suggested that NET induction is optimal at low serum concentrations (≤2%) (Fuchs et al., 2007). Recently, Neubert et al. (2019) presented a systematic literature review on studies examining NET formation *in vitro* in order to assess which medium supplements were commonly used by groups working with NETs. Indeed, they found a great heterogeneity in the media supplements used. Notably, medium without any serum or serum albumin supplement was used in the majority of the reports for production of NETs (51 and 56% of the studies on human and murine neutrophils, respectively; Neubert et al., 2019). Moreover, it was revealed that addition of fetal bovine serum (FBS) or bovine serum albumin (BSA) prevented NET formation by human neutrophils following stimulation of two commonly used NET activators, lipopolysaccharides and calcium ionophores (Neubert et al., 2019). Despite the fact that it has been known for at least 25 years that exposure to serum-free conditions induces apoptosis (O’Brien et al., 1996), several experimental steps involving serum-free conditions continue to be used. These include protocols for isolation of peripheral blood mononuclear cells (PBMC) and procedures used in every-day routine lab-work such as washing cells in phosphate buffered saline (PBS). Furthermore, protein-free solutions are prerequisites for certain cellular experiments. Several analytical assays for cell-proliferation and ROS measurements require protein-free conditions while loading cells, and transfection experiments using i.e., lipofectamine must be performed in serum-free media.

In this study, we reveal that 2–3% of cells instantly rupture and release their chromatin with preserved tertiary structure, including well-preserved lobules, when placed in protein-free cell culture media or commonly used buffers such as PBS and Hanks’ balanced salt solution (HBSS). This phenomenon is distinct from NETs and DNA release during ACD induced by osmotic or detergent lysis. Notably, this type of extracellular DNA release is not restricted to immune cells. These findings will likely have widespread implications for how cell research in general should be conducted and, more specifically, sound a cautionary note to the immunological research community to avoid unintended immune activation and erroneous interpretations in the field of extracellular traps.

## Materials and Methods

### Cells

Blood was obtained from healthy volunteers visiting the local blood center. PBMCs were isolated using Ficoll-Paque PLUS density gradient media (GE Healthcare, Uppsala, Sweden) with 2% FBS (Gibco, Invitrogen Corporation, Carlsbad, CA, United States) in all washing steps. B-cells, T-cells, and monocytes were isolated using positive selection with CD19, CD3, and CD14 MACS microbeads (Miltenyi Biotec, Bergisch Gladbach, Germany), respectively, according to the manufacturer’s instructions. Polymorphonuclear neutrophils (PMNs) were isolated from peripheral whole blood of healthy adults, after informed consent according to the recommendations of the local Research Ethics Committee of Linköping University, by gradient centrifugation using Percoll (GE Healthcare). In brief, blood was centrifuged at 1,500 × *g* before the buffy coat was collected and layered onto a Percoll gradient of 63 and 72% Percoll. Cells were centrifuged at 490 × *g*, and the PMN-containing interphase was collected. Remaining red blood cells were lysed through hypotonic lysis with double-distilled water. Lymphocyte B-cell line Nalm-6 was cultured in RPMI-1640 (Gibco) supplemented with glutamine (Gibco), penicillin-streptomycin (Gibco), and 10% FBS (Gibco). HEK 293T cells were cultured in DMEM supplemented with GlutaMAX (Gibco), glutamine, penicillin-streptomycin, and 10% FBS. Cell viability was >93% in all experiment as judged by trypan blue staining.

### Formation of Extracellular DNA With Maintained Nuclear Shape

A total of 100,000 immune cells were collected by centrifugation (300 × *g*, 5 min) and resuspended in 150 μl RPMI-1640, or other indicated solution, with or without FBS. Cells were immediately transferred to a well in a 48-well plate and incubated at 37°C and 5% CO_2_ for indicated times (between <30 s and 30 min). HEK 293T cells were detached by trypsin prior to seeding and incubation in DMEM without FBS. In certain experiments, freshly isolated PBMC were incubated in RPMI-1640 supplemented with either 10, 5, 2, or 0.5% FBS for 16 h prior to serum removal and quantification of released DNA entities. In all other experiments, cells were either used directly after isolation from blood (primary cells) or from harvesting cells growing in culture (Nalm-6).

### Visualization of Extracellular DNA

Released DNA was stained with SYTOX Green (400 nM; Thermo Fisher Scientific, Waltham, MA, United States) and visualized using Nikon Eclipse E600W fluorescence microscope equipped with a Nikon DS Ri1 digital camera. Microscopy were either conducted using live imaging by placing the plate directly below the 4× objective or after fixation of DNA on glass slides prior to microscopy (higher magnifications). Immobilization of DNA was performed by seeding 300,000 cells in 450 μl serum-free medium in wells of a 24-well plate followed by placement of a coverslip on top of the liquid surface for 5 min. Subsequently, coverslips were mounted in fluorescent mounting medium (Agilent Dako, Santa Clara, CA, United States). A schematic illustration of the procedure is shown in Supplementary Figure S1. For analysis of DNA distribution in a well of a 48-well plate containing 100,000 Nalm-6 cells in 150 μl PBS/well, a widefield Z-stack covering the whole focal volume of the liquid (from bottom of well; 776 μm, 325 stacks) was obtained using a LM Leica DMi8 microscope with a 20×/0.4 objective. Deconvolution and the maximum intensity projection were done using Huygens Imaging Software (SVI, Hilversum, Netherlands).

### DNase Treatment

DNase treatment was performed by adding 4 U/ml DNase I (Sigma-Aldrich, Saint Louis, MO, United States), 2.5 mM MgCl_2_ and 0.5 mM CaCl_2_ to the wells containing released DNA and incubated for 37°C for 60 min. After SYTOX Green staining, samples were subjected to live imaging using 4× objective.

### Quantification of DNA-Release

For quantification of DNA-releasing ability in different samples, stained DNA entities were visualized by live imaging (4× objective). Three images were obtained from different positions in each well. Some degree of overlap occurred. The number of released DNA entities were counted using the software’s in-built object count feature (NIS Elements BR 4.20, Nikon). For quantification of DNA-releasing cells, cells were seeded in 150 μl PBS as described above and incubated for 30 min. Extracellular DNA was stained with SYTOX Green and visualized using live imaging. Multiple images were recorded using the Grab Large Image tool in NIS Elements BR 4.20 (Nikon) until the entire well was captured. Subsequently, the number of released DNA entities was manually counted and related to the number of cells seeded. Experiments were carried out three times for each condition at different occasions. When analyzing the impact of additional PBS washes on the ability to release DNA entities, Nalm-6 cells were washed in duplicates. Following the last wash, one sample was resuspended in PBS and used for quantification of released DNA entities while the other sample was resuspended in FBS containing PBS and used to determine the number of cells used in the quantification.

### Inhibition of DNA Release

A total of 100,000 Nalm-6 cells were seeded in RPMI-1640 containing either of the below listed substances at different concentrations. Following a 30 min incubation, DNA was stained with SYTOX Green and quantified as described above. The number of DNA entities in the non-inhibited control was regarded as maximum DNA release (100%). The following substances and concentrations were used for inhibition studies presented in the work: FBS (0.01–10%), BSA (0.001–1 mg/ml; Roche, Basel, Switzerland), polyethylene glycol (PEG) (m.w. 200, 400, 600, 1,000, 4,000, 6,000, and 8,000 g/mol; 2.5 nM, 2.5 μM, and 2.5 mM), and Pluronic F-68 (0.0001–0.01%; Thermo Fisher Scientific). All experiments were performed at least on three different occasions.

### Cell Counting

A total of 100,000 Nalm-6 cells were seeded in microcentrifuge tubes in PBS with or without supplement of FBS, BSA, PEG, or Pluronic F-68 and incubated for 5 min at 37°C. Following resuspension by pipetting, cells were counted in a TC10 automated cell counter (Bio-Rad, Hercules, CA, United States). As controls, samples incubated in PBS without additives for 5 min to which indicated substances were added prior to resuspension and cell counting were also included. Samples were counted twice and the experiment was repeated five times for every condition.

### Time Titration

A total of 100,000 cells (Nalm-6 and PBMC) were resuspended in 150 μl PBS and incubated in wells of a 48-well plate. BSA to a final concentration of 1 mg/ml was added to the wells at different time points between 5 s and 30 min. For the 0 s time point, cells were resuspended in PBS and then directly added into a solution of BSA. As control, cells were resuspended in BSA (1 mg/ml) containing PBS. Following staining with SYTOX Green, the number of released DNA entities was quantified as described above as well as by measuring fluorescence using a fluorescent plate reader (excitation wavelength: 485 nm/emission wavelength: 535 nm. Spark 10M, Tecan, Männedorf, Switzerland). The experiment was repeated six times for the Nalm-6 cell line and in total eight times for PBMC isolated from two cell donors.

### Literature Review

Between February 22 and March 5, 2019, a systematic literature review was conducted. The following term was used to search articles in PubMed:

human PBMC (“2018/01/01”[Date–Publication] : “2018/12/31”[Date–Publication])

This generated 477 articles in total. Of them, 101 articles were not included in the analysis due to lack of subscription, non-English language, video articles, review articles without experimental procedures, or non-relevant articles (e.g., PBMC not related to peripheral blood mononuclear cells). In the remaining 376 articles, all Materials and Methods sections were reviewed and information about PBMC isolation procedure, protein-free washing steps, and experiments carried out in protein-free conditions were collected. A few studies involving non-human PBMC that ended up in the search result were included as well.

### Staining of DNA Along With Cellular Components

Immunolabeling of cellular proteins was conducted using Nalm-6 extracellular DNA immobilized on coverslips. DNA was fixed in 4% formaldehyde for 15 min at room temperature followed by three washes in PBS. Blocking was performed at room temperature for 60 min in PBS containing 5% FBS and 0.3% Triton X-100. Slides were incubated for 40 min with primary antibodies (Supplementary Table S1) diluted in PBS with 1% BSA and 0.3% Triton X-100 at room temperature. Following three washes in PBS, secondary antibodies in PBS with 1% BSA and 0.3% Triton X-100 were added for 30 min at room temperature. Slides were washed three times with PBS and DNA was stained with DAPI prior to mounting in fluorescent mounting medium. Staining of mitochondria and cytoplasmic membranes was done using Mitotracker Green FM (Invitrogen) and CellBrite Cytoplasmic Membrane Dye (Biotium, Fremont, CA, United States), respectively. Prior to seeding in PBS, Nalm-6 cells, 1 × 10^6^ cells in 1 ml PBS/1 mg/ml BSA, were stained with 160 nM Mitotracker Green FM or 5 μl CellBrite for 20 min at 37°C. Following two washes in PBS/1 mg/ml BSA, cells were resuspended in PBS and seeded in wells of a 24-well plate (300,000 cells in 500 μl). Coverslips were immediately placed on top of the liquid surface to trap DNA also during the initial phases of extrusion. After 5 min, coverslips were washed and stained with DAPI in PBS and mounted in fluorescent mounting medium. For pedagogical purposes, extracellular DNA is shown in green throughout the article.

### Nocodazole Treatment of Nalm-6 Cells

To increase the number of cells in G2/M Nalm-6 cells (1 × 10^6^ cells/ml) were treated with nocodazole (100 ng/ml; Sigma-Aldrich) for 15 h in complete cell culture medium. Following washes in fresh cell culture medium, cells were resuspended in PBS and seeded in wells of a 24-well plate. DNA was immobilized on coverslips and processed as above.

## Results

### DNA Extrusion From Cells in Serum-Free Conditions

**FIGURE 1:**
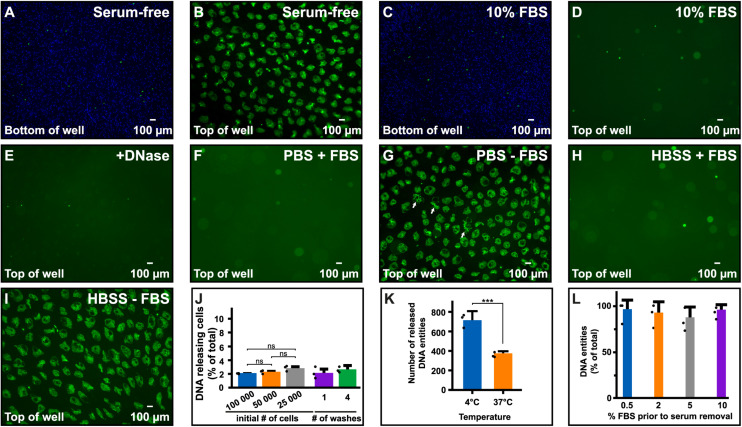
Cells release their DNA in serum-free media. **(A–D)** Microscopy images using a 4× objective directly over the plate in which Nalm-6 cells were seeded in medium with or without FBS and stained with Hoechst 33342 (blue). Following incubation for 30 min, SYTOX Green nucleic stain (green) was added to stain extracellular DNA and necrotic cells. **(A)** Nalm-6 cells at the bottom of the well seeded in serum-free medium. **(B)** Extracellular DNA in supernatants at the top of the same well as in panel **(A)**. **(C)** Nalm-6 cells in serum containing medium at the bottom of the well. **(D)** Extracellular DNA at the top of the same well as in panel **(C)**. **(E)** Extracellular DNA in supernatants after treatment with DNase. **(F–I)** Nalm-6 cells in PBS and Hanks’ balanced salt solution (HBSS) with **(F,H)** or without **(G,I)** 10% FBS using a 4x objective. Arrowheads in panel **(G)** highlight irregular shaped DNA. **(J)** Number of Nalm-6 cells, expressed as percentage of total, releasing DNA extracellularly when seeded in serum-free medium. Cells were seeded in 150 μl to study the impact of cell density and washing on the release. All released DNA entities were counted and related to the total number of cells in the experiment. *N* = 3. **(K)** Release of DNA from Nalm-6 cells incubated for 30 min in PBS at two different temperatures. Data shows the mean of three independent experiments and represents the number of DNA entities observed in three individual images recorded from different positions in each sample. **(L)** Quantification of DNA release after acclimatizing cells to different concentrations of FBS for 16 h prior to serum removal. No significant differences were observed. Statistical analyses were done using unpaired *t*-test **(K)**, ***P < 0.001, or one-way ANOVA followed by Turkey’s multiple comparisons test **(J,L)**. Filled circles in panels **(J–L)** represent the mean in each individual experiment.

While studying extracellular release of DNA from immune cells using the pre-B-cell line Nalm-6, we noticed large DNA entities in the supernatant when cells were exposed to serum-free medium, but not when FBS was present (Figures 1A–D). Notably, the extracellular DNA emerged at the top of the liquid while cells were at the bottom (Figures 1A,B). These released structures contained deoxynucleotides as confirmed by their disappearance upon DNase treatment (Figure 1E). DNA release occurred also in physiological buffers commonly used for washing cells, e.g., PBS and HBSS (Figures 1F–I). A schematic illustration of how the extracellular DNA was visualized is shown in Supplementary Figure S1.

**FIGURE 2:**
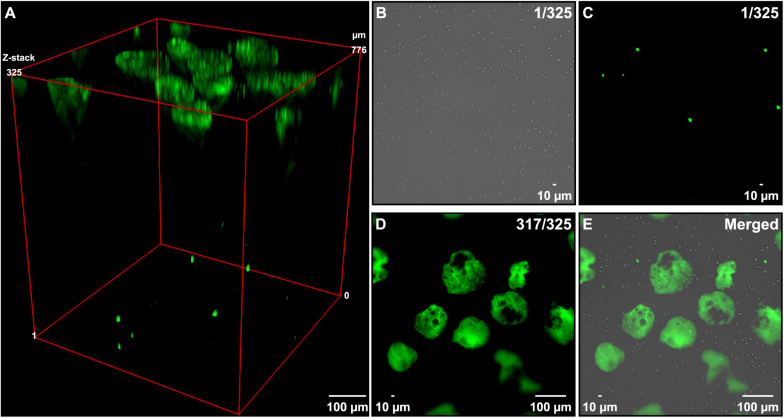
Axial assessment of DNA entities. Nalm-6 cells in serum-free PBS were placed in a 48-well plate and the axial localization of the released DNA was determined by microscopy. **(A)** Maximum intensity Z-projection of 325 widefield Z-stacks covering the whole focal volume of the liquid in a well (776 μm). Green fluorescens origin from extracellular DNA stained by the cell membrane-impermeant dye SYTOX Green nucleic stain. **(B)** Bright-field image of cells in Z-stack 1 out of 325. **(C)** Fluorescent image of the corresponding image shown in panel **(B)** representing dead cells. **(D)** Image of stack 317 out of 325 recorded in the fluorescent channel showing the extracellular DNA released when placing cells in serum-free solutions. **(E)** An overlay image obtained by combining images shown in panels **(B–D)**.

By visually comparing the number of extracellular DNA entities (Figure 1B) with the number of cells in the same view (Figure 1A), it seemed like only a fraction of the cells released their DNA under these conditions. To quantify the percentage of cells releasing their DNA, we incubated a known number of Nalm-6 cells (e.g., 25,000, 50,000, and 100,000 cells) in serum-free medium in a 48 well plate for 30 min at 37°C, counted all of the released DNA entities and related this number to the total number of cells used in the experiment. In these experimental conditions, we observed that 2–3% of Nalm-6 cells released their DNA (Figure 1J). Only a minor cell density dependence was observed and additional washes in serum-free solutions did not affect the ability of cells to release DNA entities (Figure 1J). However, DNA release was more prominent at 4°C compared to 37°C (Figure 1K). Allowing cells to acclimatize to lower serum conditions for 16 h did not significantly affect the ability to release DNA (Figure 1L). Pipetting did not significantly impact the number of released DNA entities (Supplementary Figures S2A–F). After release, DNA entities persisted in the medium for at least 72 h when left in serum-free medium (Supplementary Figures S2G–I). To confirm the surface location and assess the axial distribution of the released DNA, we performed a widefield Z-stack analysis directly in the plate. Strikingly, released DNA entities appeared in one level at the liquid surface, while viable and necrotic cells remained at the bottom of the well (Figures 2A–E).

**FIGURE 3:**
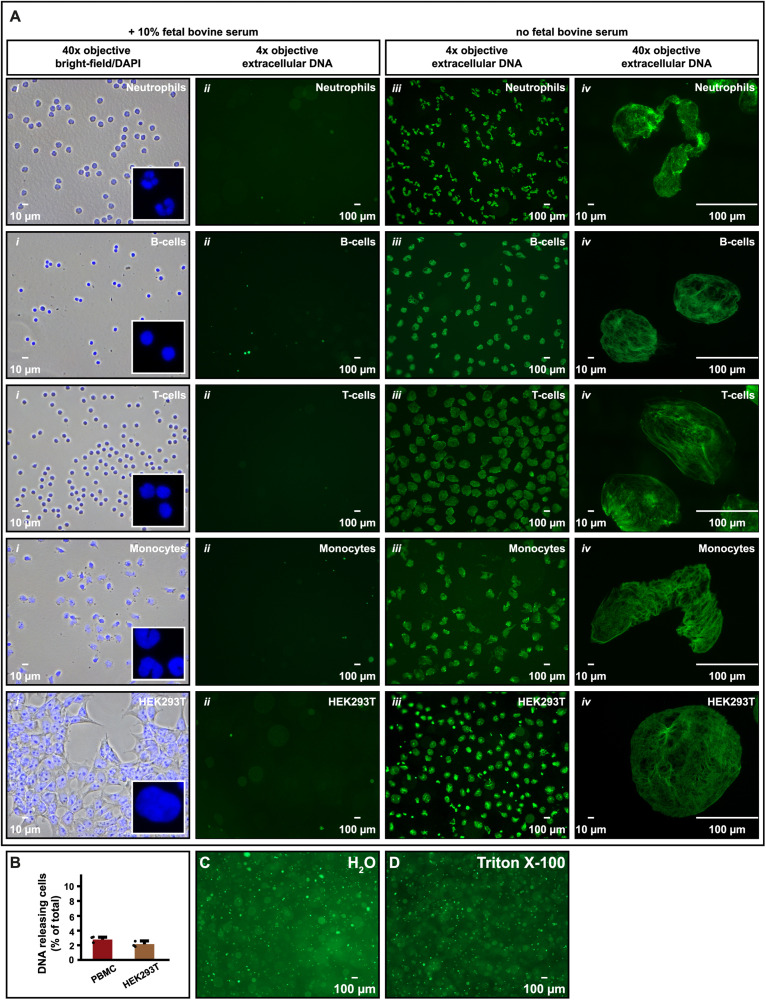
DNA release is not restricted to hematopoietic cells. **(A)** Microscopy images of human neutrophils, B-cells, T-cells, and monocytes as well as HEK293T cells in cell culture medium with or without 10% FBS as supplement. In *i*, cells were fixed and stained with DAPI to visualize the size and shape of nuclei in physiological conditions for comparison (*i*; 40× objective). To further highlight the shape of the nuclei, parts of the images were enlarged (white box). *ii* and *iii* are microscopy images (4× objective) of extracellular DNA in supernatants released from cells after 30 min in FBS containing (*ii*) or FBS deprived (*iii*) cell medium after staining with SYTOX Green nucleic stain. In *iv*, released DNA in *iii* was captured on a glass slide and imaged using the same magnification as in *i* (40× objective) to highlight the shape and size of the released DNA. **(B)** Number of PBMC and HEK293T cells, expressed as percentage of total, releasing DNA extracellularly when seeded in serum-free medium. All released DNA entities were counted and related to the total number of cells in the experiment. *N* = 3. **(C,D)** Extracellular DNA released from Nalm-6 cells seeded in deionized water **(C)** or in 10% Triton X-100 in PBS **(D)** and stained with SYTOX Green. Microscopy images of extracellular DNA in panels **(C,D)** were obtained using 4× objective.

We used similar experimental conditions to study if also other cell types responded to serum deprivation in the same manner. Human primary immune cells (neutrophils, B-cells, T-cells, and monocytes) were isolated and seeded in RPMI-1640 without FBS as supplement. Also, in these samples we observed extracellular DNA floating in the medium but not when 10% FBS was present (Figure 3A, *ii*, *iii*). This was also seen for the non-immune cell HEK293T after trypsinization and resuspension in serum-free medium (Figure 3A, *iii*). It was however, not observed for HEK293T while still adhering to the cell culture plate (not shown). Like Nalm-6, the number of DNA-releasing PBMC and HEK293T was quantified to be 2–3% (Figure 3B). DNA entities at the liquid surface could be immobilized on glass slides and used for microscopy at higher magnifications than microscopy directly in the medium allowed (Supplementary Figure S1). This revealed that, unlike DNA released by cytolysis or detergents (e.g., Triton X-100) (Figures 3C,D), the microscopic appearance of the extruded DNA resembled the shape of the corresponding original cell nucleus, although ∼10 times enlarged. This was most clearly recognizable for neutrophils and monocytes with their lobulated nuclei (Figure 3A, *i*, *iv*).

### Prevention of Rapid Cell Lysis

**FIGURE 4:**
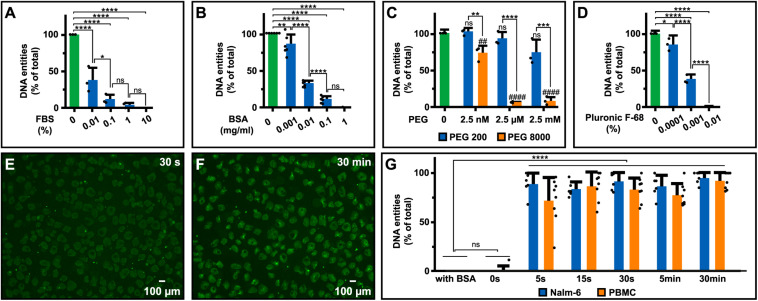
Extracellular DNA upon various treatments. Impact of **(A)** FBS (*N* = 3), **(B)** BSA (*N* = 6), **(C)** PEG (*N* = 3), and **(D)** Pluronic F-68 (*N* = 3) on DNA release from Nalm-6 cells in RPMI-1640 with additives of indicated concentrations for 30 min prior to staining of extracellular DNA and microscopy analyses. The amount of DNA entities in the serum-free control sample was considered 100% to which all samples were compared. **(E,F)** Extracellular DNA released from 100,000 Nalm-6 cells visualized in supernatants from the same sample after less than 30 s **(E)** and 30 min **(F)** upon serum withdrawal imaged using 4× objective. **(G)** Assessment of the timeframe for DNA release upon serum removal. Nalm-6 cells (*N* = 6) and PBMC (*N* = 8) were incubated in PBS for indicated times before BSA to a final concentration of 1 mg/ml was added. The time point with the highest amount of released DNA entities in each experiment was considered 100% to which all samples were compared. Filled circles in panels **(A–D,G)** represent the mean for each individual experiment and error bars represent standard deviations calculated using the mean for all biological replicas **(A,B,G)** or all included technical replicas for the three independent experiments **(C,D)**. Statistical analyses were done using one-way ANOVA followed by Turkey’s multiple comparisons test **(A–D)** and Dunnett’s multiple comparisons test **(G)**. *P < 0.05, **/^##^*P* < 0.01, ***P < 0.001, ****/^####^*P* < 0.0001.

DNA-release could be prevented by FBS, BSA, PEG, and Pluronic F-68, in a concentration-dependent manner (Figures 4A–D). Inhibition by PEG also showed a clear molecular weight dependence with PEG ≥ 4000 having the strongest effect (Figure 4C and Supplementary Table S2). Addition of 0.3 M sucrose to RPMI-1640, which increases osmolality of RPMI-1640 medium from 260 to 300 mOsm/kg (e.g., isotonic) to ∼600 mOsm/kg, could not prevent DNA-discharge (Supplementary Figure S2J). In comparison, addition of PEG (m.w. > 1,000 Da) at μM concentrations only marginally affect osmolality (<1 mOsm/kg) but still exerted a protective effect (Supplementary Table S2). Next, we analyzed how soon after serum deprivation DNA could be observed. The release occurred very rapidly, extracellular DNA entities were visible already after <30 s of serum removal (Figures 4E,F). In fact, DNA was released faster than binding of SYTOX Green occurred, hampering an exact determination of the timeframe in which the discharge occurs.

To circumvent this problem, cells (e.g., PBMC and Nalm-6 cells) were incubated in PBS at different lengths of time (5 s–30 min) before addition of albumin to prevent further release. The amount of released DNA was quantified by two different methods: (1) counting of extracellular DNA entities using microscopy (Figure 4G) and (2) measurements of SYTOX Green fluorescence intensity in wells using a plate reader (Supplementary Figure S2K). Both methods were congruent with results showing that most DNA entities are released within the first seconds after serum removal.

### Serum-Free Conditions Introduce Unintentional Bias

**FIGURE 5:**
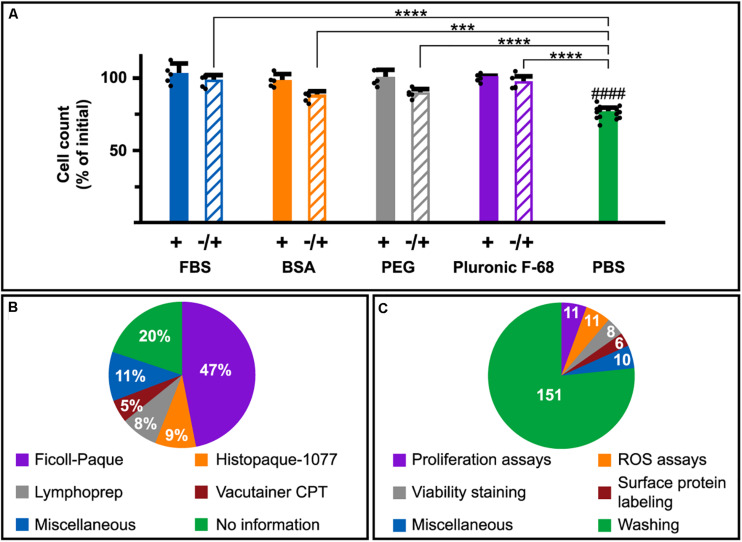
DNA-release promoting conditions in immunological research. **(A)** Counting of Nalm-6 cells incubated in PBS with (filled bars) or without (green bar) 10% FBS, 1 mg/ml BSA, 2.5 mM PEG8000, or 0.01% Pluronic F-68 for 5 min prior to cell counting. Samples incubated with PBS without supplement for 5 min followed by addition prior to cell counting are seen as striped bars. The results are expressed as percentages of the initial number of cells and represent the mean of five independent experiments. For the PBS control (green bar), *N* = 15. The mean of each experiment is represented by a filled circle. Statistical analysis was done using one-way ANOVA followed by Turkey’s multiple comparisons test. ***P < 0.001, ****P < 0.0001. #### indicates significant differences (*P* < 0.0001) toward samples incubated in PBS with supplements (+; filled bars). **(B)** Pie charts of PBMC isolation methods used in 376 reviewed relevant articles. Figures represent percentages of all considered publications. **(C)** Pie charts of experimental procedures involving protein-free conditions used in the 376 studied articles. Figures represent the absolute numbers of publications using indicated experimental procedures in protein-free conditions while the slices represent percentages of all articles describing usage of protein-free conditions at any step.

Besides the actual loss of cells due to cell death, we also observed that a 5 min incubation of cells in PBS displayed an apparent decrease of cell numbers by approximately 25% (Figure 5A; green bar) compared to cells maintained in PBS supplemented with FBS, BSA, PEG, or Pluronic F-68 (Figure 5A; filled bars). This does not correlate with the number of DNA releasing cells (2–3%; Figure 1J). Therefore, we investigated whether this observation was an actual cell elimination or an apparent loss due to biases in cell counting. Addition of FBS, BSA, PEG, or Pluronic F-68 after the 5 min PBS incubation, but prior to cell counting, resulted in a significant reappearance of cells compared to samples in PBS only (Figure 5A; striped bars).

For an overview and in order to broaden our perception of how common it is to maintain immune cells in protein-free solutions, we reviewed the PubMed literature published in 2018, in which experiments on PBMC, in some aspects, were performed (for details see section “Materials and Methods”). 301 of the 376 included articles provided information regarding isolation procedures. 230 of those (76%) used methods containing preparation steps involving washes in protein-free solutions according to the manufacturer’s instructions [e.g., Ficoll-Paque (GE-Healthcare), Histopaque-1077 (Sigma-Aldrich) and Vacutainer CPT (BD Biosciences)] (Figure 5B). Providers of Lymphoprep (Stemcell Technologies) do not explicit suggest protein-free solutions. Instead the protocol advices to wash mononuclear cells “*once with medium*” which is open for interpretations. Worth highlighting is that five of the articles were method-oriented, providing instructions for PBMC isolation aimed for i.e., biobanking and immune system studies. All those papers included steps involving resuspension of PBMC in protein-free solutions. Only five articles (1.3%) claimed the use of FBS or BSA supplemented solutions (including AIM V serum-free medium) for washing of PBMC during isolation. 151 articles (40%) clearly stated that protein-free solutions were used for cell washing during different steps of the experimental procedure and in 46 studies (12%) protein-free buffers were used as a part of the experiment (i.e., viability, proliferation, and ROS assays) (Figure 5C).

### Extracellular Chromatin as a Tool to Study DNA Organization

**FIGURE 6:**
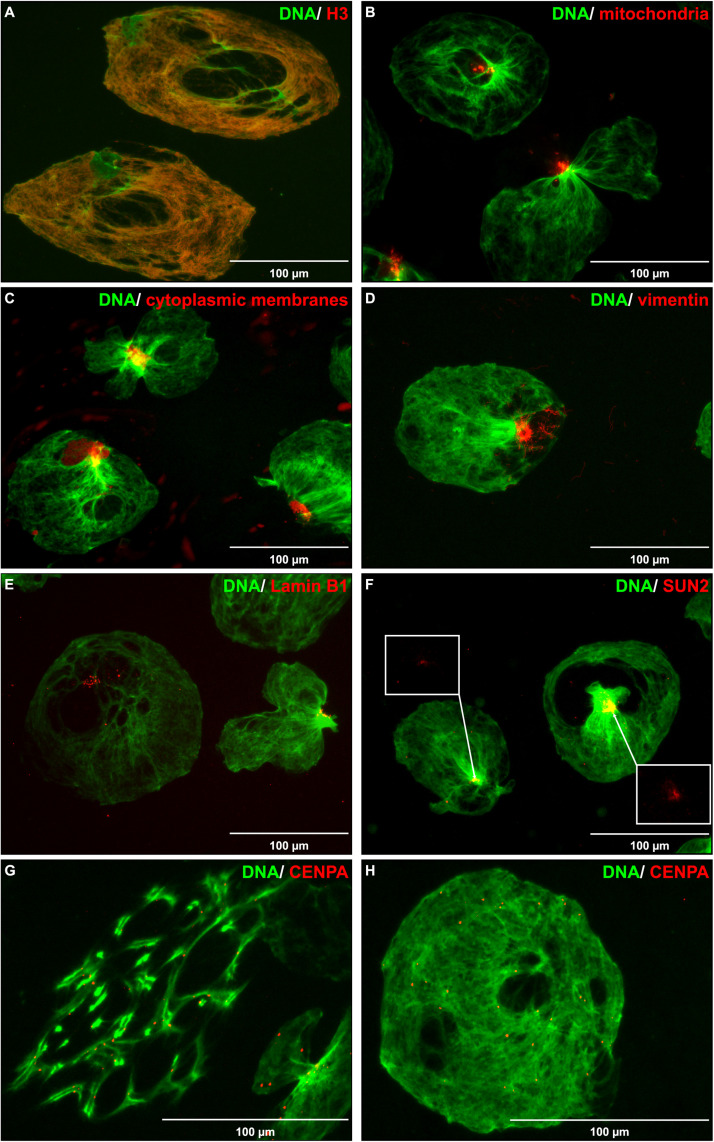
Staining of DNA along with cellular components. Microscopy images (40× objective) of extracellular DNA (green) released from Nalm-6 cells, immobilized on glass slides, and stained for different cellular components (red). **(A)** Immunostaining of Histone H3. **(B)** Staining of mitochondria using Mitotracker. **(C)** Membrane labeling using CellBrite Cytoplasmic Membrane Dye. **(D)** Immunostaining of Vimentin. **(E)** Immunostaining of Lamin B1. **(F)** Immunostaining of SUN2. **(G)** Immunostaining of Centromere protein A (CENPA) after nocodazole treatment of cells prior exposure to serum-free conditions. **(H)** Immunostaining of CENPA without prior nocodazole treatment. Scale bars equal 100 μm. In panel **(F)**, white boxes highlight SUN2 staining.

In order to make use of the enlarged DNA and study chromatin composition and shape, released DNA entities were immobilized on coverslips and subjected to staining of cellular components using immunolabeling and organelle targeted dyes. Core histones (H2B, H3, and H4), not linker histone H1, aligned perfectly with the released DNA (Figure 6A and Supplementary Figures S3A–C). In contrast, staining of mitochondria, cytoplasmic membranes, and the cytoskeletal protein vimentin all appeared in distinct, confined, spots (Figures 6B–D). Nuclear membrane associated proteins, e.g., Lamin B1 and SUN2, did not align with the DNA but appeared at a more confined area (Figures 6E,F), similar to the staining pattern of vimentin and mitochondria. Occasionally, we observed irregular chromatin structures reminding of chromosomes in metaphase rather than resembling the shape of the nucleus (Figure 1G, arrows). To test this hypothesis, the number of Nalm-6 cells in G2/M phase were increased by nocodazole treatment for 15 h prior to serum removal. Nocodazole treatment considerably increased the proportion of chromosome-like chromatin entities (Supplementary Figure S3D). Immobilization of discharged chromatin on coverslips and immunostaining of Centromere protein A revealed highly organized sister chromatids within a restricted area (Figure 6G). Centromere protein A could also be observed in the non-mitotic chromatin at evenly spread positions (Figure 6H).

## Discussion

This study reports on a previously undescribed rapid lytic cell death accompanied by chromatin release. It shares many features of an ACD, but there are also signs of RCD. While osmotic and detergent induced cell deaths, two examples of ACDs, result in plasma membrane rupture, the DNA is not released in a similar manner as it is upon serum removal. To exclude the possibility that cells ruptured because of sudden changes in serum concentrations, we allowed cells to acclimatize at various FBS concentrations overnight. All pretreatments gave similar results. In our experimental setup, only 2–3% of the cells ruptured nuclear and plasma membranes with concomitant release of chromatin entities while the vast majority of cells did not, suggesting a heterogenous/bimodal phenotype response. This is in sharp contrast to cells that are exposed to osmotic or detergent induced cell lysis, where 100% of cells die. However, since we noticed that cold could influence the number of released chromatin entities, it is likely that other factors might impact on the amount of bursting cells as well. Our observations suggest an underlying mechanism controlling which cells are to be lysed. It is possible that this might be a protective procedure and that the fate of a dying cell is to protect the majority of the cells from rupture by release of cellular components shielding cells similarly to FBS, albumin, PEG, and Pluronic-F68. Interestingly, strengthening this hypothesis are the results obtained after washing of the cells more than once with PBS where 2–3% of the cells again extruded their DNA, implicating that it maybe is not a specific cell subtype that releases its DNA. However, this hypothesis has to be proven. The results obtained using PEG are comparable with those reported by Vanderpol et al. (1995) in the 1990s, when PEG was used as an additive to avoid cell death due to shear stress induced by sparging while bubbling air into hybridoma cell cultures in serum-free medium for antibody production. Moreover, shear stress was used to explain erroneous viability measurements of cells in PBS during cell counting procedures (Chen et al., 2017). Similar, although only a small fraction of all cells dies and discharge their chromatin when placed in serum-free conditions, we found a 25% decrease in cell number upon cell counting in PBS. Indeed, the decrease constitutes an apparent loss of cells since addition of the above-mentioned compounds immediately prior to counting, but after a 5 min incubation in PBS, reversed cell loss. We believe that FBS, BSA, PEG, and Pluronic F-68 act as surfactants to prevent cells from being ensnared in large adherent DNA and lost during pipetting, generating an apparent loss of cells and erroneous cell density assessment during the counting procedure. Erroneous viability measurements can introduce secondary failures that have impact on experimental approaches and interpretations. Thus, it is important to select a correct diluent to assure accurate and representative assessment of cell number.

Although cells in physiological conditions are not exposed to an environment devoid of macromolecules (i.e., albumin), this release may also be triggered by other molecules/stresses not known at present. The actual trigger mechanism for cell lysis and chromatin-release in serum-free solutions has still to be confirmed. Nevertheless, our results clearly show that it is not an effect of mere osmosis. Since this way of dying is distinct from other types of ACDs, involves some degree of regulation, and does not match the criteria for any type of previously described RCD (Galluzzi et al., 2018), we propose that future studies refer to this event as *peniaptosis* (from the Greek word penia, meaning lack of/shortage of necessary goods).

Despite that exposure to serum-free conditions has been known for a long time to induce apoptosis (O’Brien et al., 1996), several experimental steps involving serum-free conditions, in which according to our results a few percent of the cells will die and release their chromatin extracellularly, continue to be used. Highly relevant in immunological research, the released chromatin may also activate leukocytes since extracellular chromatin is well-recognized as a damage associated molecular pattern (Paludan and Bowie, 2013). The most frequent experimental step conducted in protein-free solution used in everyday lab-bench work is probably short washes in balanced salt solutions (i.e., PBS, HBSS, and saline). Since we found chromatin entities to be discharged within seconds, and to occur both at 4°C and 37°C, this concerns many researchers across different areas. Indeed, our literature review revealed that 40% of the studies clearly stated that protein-free solutions were used for cell washing during different steps of the experimental procedure. The actual prevalence is presumably higher since several studies mentioned washing-steps without providing further information regarding the buffer used or even failed to mention washes, although it could be anticipated from the context.

Although awareness of rapid chromatin discharge and cell loss is of significance in the broader sphere of cell research, this knowledge is of utmost importance in the field of extracellular DNA research. According to definitions, NETs are restricted to cells of hematopoietic derivation and dependent on ROS (Galluzzi et al., 2018). As shown here, chromatin release in serum-free environments is not restricted to hematopoietic cells. We were able to detect an increase in ROS levels after 30 min in serum-free medium, however, at this time-point the chromatin was already formed (not shown). We were not able to detect any rapid changes in ROS levels during the initial seconds it takes for the chromatin entities to be released. The fact that over 50% of all experiments on NETs *in vitro* are performed in serum-free conditions, as reported by Neubert et al. (2019), is troublesome. It is also worrying that two commonly used NET activators, lipopolysaccharides and calcium ionophores, were reported to promote NET formation in human neutrophils only in the absence of FBS or BSA (Neubert et al., 2019). These facts raise concern in the entire research field and further highlights the need for more standardized methods for NET formation as has been requested by the NET community itself (Boeltz et al., 2019). Moreover, the newly gained knowledge from the present study implies that some of the reports on NET-formation in serum-free media most likely need to be re-evaluated. For example, a Nature Medicine article reporting that CXCL8 efficiently could induce NET-formation *in vitro* (Marcos et al., 2010) was retracted. The authors found that the cell culture condition used (RPMI-1640 medium in the absence of albumin or serum) allowed nonspecific neutrophil activation (Marcos et al., 2011). With that in mind, and in the light of our present data, we sound a cautionary note to the immunological research community and recommend researchers within the field of NETs to be very cautious while studying immune cell activation and generation of extracellular traps in serum-free conditions.

The enlarged expelled chromatin appears to maintain many of its interactions and thus provides a rapid tool for analysis of chromatin organization and genome shape. Intriguingly, unlike DNA released during NET-formation and lysis by osmosis or detergents, the microscopic appearance of the enlarged extruded chromatin resembled the shape of the corresponding cell nucleus. This was most apparent for neutrophils and monocytes with their lobulated nuclei. We hypothesize that this phenomenon can be utilized in studies of genome organization and shape. In a Comment in Developmental Cell on super-resolution imaging of chromatin, Finn et al. (2016) claim that elucidating chromatin’s 3D shape is critical to understanding its function, but that the fine structure of chromatin domains remains poorly resolved. By immobilizing released chromatin entities on coverslips, we managed to visualize the released chromatin along with stained cellular components at a remarkable resolution. While histones and centromere protein A aligned with the DNA, other cellular components, including cytoplasmic membrane and intracellular membrane structures as well as mitochondria and cytoskeleton proteins appeared in distinct, confined, spots. Presumably, this represents the original cell from which the chromatin was released. It has been hypothesized that the shape of the nucleus dictates the arrangement of chromatin, although the question whether it is the other way around has been raised (Walters et al., 2012). However, nuclear membrane associated proteins, e.g., Lamin B1 and SUN2, did not align with the DNA but appeared at a more confined area, similar to the staining pattern of vimentin and mitochondria, implicating disruption also of nuclear membrane during release. Thus, absence of inner nuclear membrane associated proteins encapsulating the extracellular DNA suggests an inherited ability of chromatin to maintain/determine its shape rather than being shaped by the surrounding envelope. This ability seems to be preserved also during mitosis as we observed discharged chromatin entities organized as elongated threads reminiscent of chromosomes in metaphase rather than resembling the shape of the nucleus. Thus, immobilizing released DNA entities on coverslips can be employed to study chromosome formation/organization during mitosis using microscopy as well.

Acknowledging that genome shape relates to genome functioning (Denker and de Laat, 2016), genome structure is of high research interest. Recently, the 4D nucleome project with the goal of gaining deeper mechanistic insights into how the nucleus is organized was launched (Dekker et al., 2017). Several projects within this network aim to map chromosome positioning, colocalized DNA regions, interactions between specific genomic loci, and chromatin interactions with specific proteins (Dekker et al., 2017). Considering that, within minutes upon serum deprivation, it is possible to produce chromatin which seems to maintain many of its interactions also after extracellular release, we encourage researchers in this field to evaluate the herein described method and utilize the enlarged DNA entities for such studies.

In summary, besides proposing a tool to study chromatin interactions, our findings forward an important message on how cells should be handled throughout all steps in experimental cell research in order to avoid introduction of bias due to cell death and cell elimination or unintended activation of signaling pathways in immune cells via DNA-binding sensors. We also report a cell death that differ from accidental death and does not match the criteria for a previously described RCD and therefore propose this, when its mechanism is elucidated, to be called *peniaptosis* owing to its initial discovery during serum deprivation conditions.

## Data Availability Statement

The raw data supporting the conclusions of this article will be made available by the authors, without undue reservation, to any qualified researcher.

## Ethics Statement

Polymorphonuclear neutrophils (PMNs) were isolated from peripheral whole blood of healthy adults, after informed consent according to the recommendations of the local Research Ethics Committee of Linköping University.

## Author Contributions

GS and BI conceived the study. BI performed the experiments. GS, DA, AR, and BI analyzed the data. DA and BI isolated primary immune cells and performed the statistical analysis. GS, AR, and BI wrote the manuscript. All authors approved the final version of the manuscript.

## Conflict of Interest

The authors declare that the research was conducted in the absence of any commercial or financial relationships that could be construed as a potential conflict of interest.

## Abbreviations

ACD: accidental cell death
HBSS: Hanks’ balanced salt solution
NET: neutrophil extracellular trap
PBMC: peripheral blood mononuclear cells
PEG: polyethylene glycol
RCD: regulated cell death
ROS: reactive oxygen species.
