# Supplementary material for: Sizing Up Extracellular DNA: Instant Chromatin Discharge From Cells When Placed in Serum-Free Conditions
Source: Front Cell Dev Biol. 2020 Jul 22;8:634. doi: 10.3389/fcell.2020.00634 (PMC7387414; doi:10.3389/fcell.2020.00634)
Supplement: Supplementary file 1 [file Data_Sheet_1.pdf]

## Supplementary Material

Spyrou et al.,

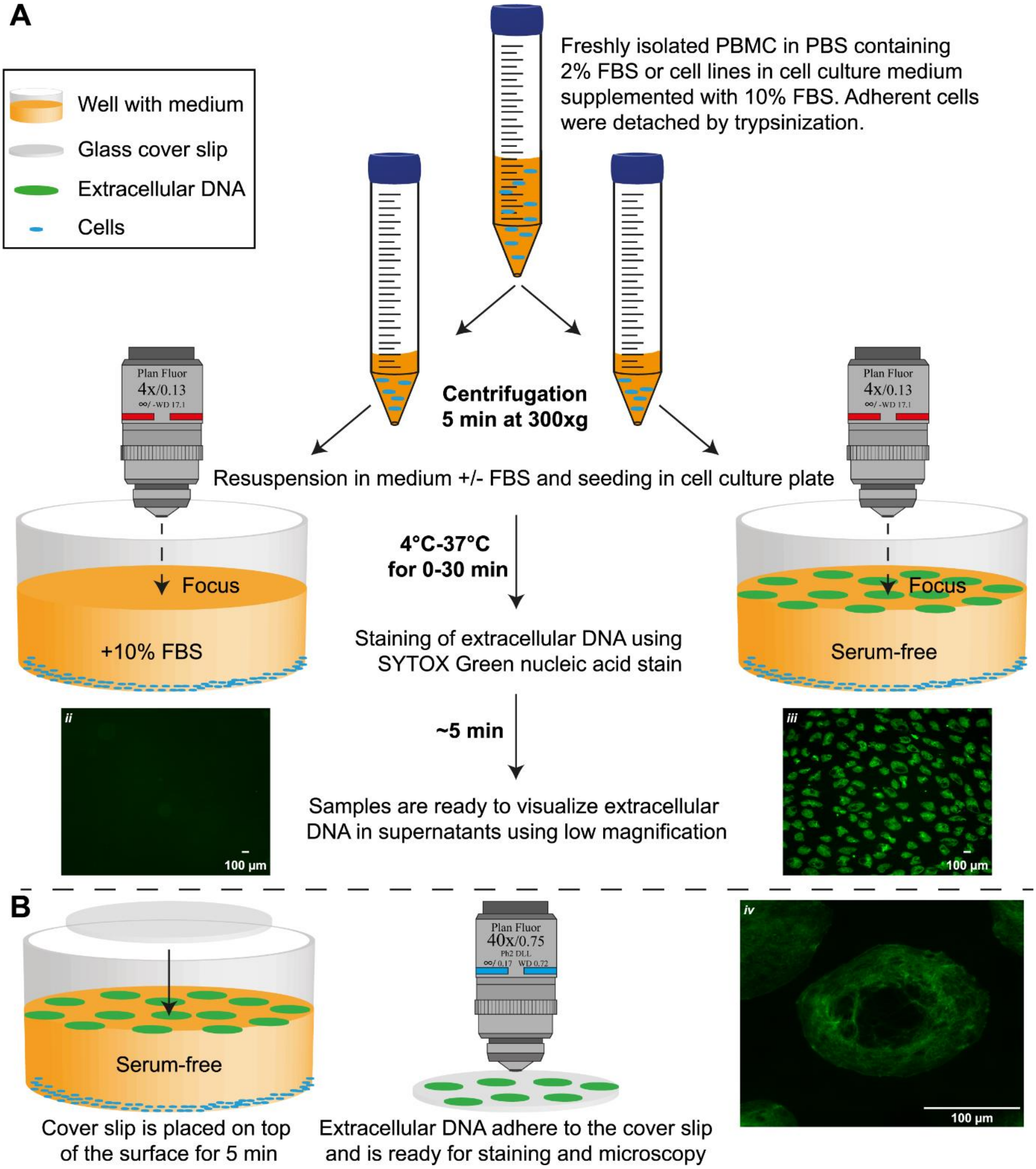


FIGURE. S1. **Overview of sample preparation.** Schematic illustration of the procedure for preparation of enlarged extracellular DNA entities. We used either PBMC, freshly isolated from healthy blood donors using Ficoll-Paque density centrifugation and resuspended in PBS containing 2% FBS, or cell lines growing in cell culture medium (e.g. DMEM or RPMI-1640 depending on the cell line) supplemented with 10% FBS. Adherent cells were detached by trypsinization. Sample was split in two, centrifuged, and resuspended in medium (i.e. cell culture medium, PBS or HBSS) with or without FBS or bovine serum albumin and seeded in wells of cell culture plates (48 well plates for A and 24 well plates for B). Following incubation for indicated times, extracellular DNA was stained by SYTOX Green nucleic acid stain and used for analysis by microscopy. The extracellular DNA was visualized directly in wells by using an objective with low magnification directly over the well with focus close to the liquid surface (A). This procedure was used to obtain images presented in Figure 1 *ii*, *iii*; Figure 2A-D, Figure 4A-D and Figure 5E, F. Alternatively, a glass cover slip was placed on top of the liquid surface for 5 min to allow

extracellular DNA to adhere to the glass before the cover slip was removed, washed, and mounted prior to microscopy analyses using higher magnifications (B). This procedure was used to obtain images presented in Figure 1 *iv* and Figure 7.

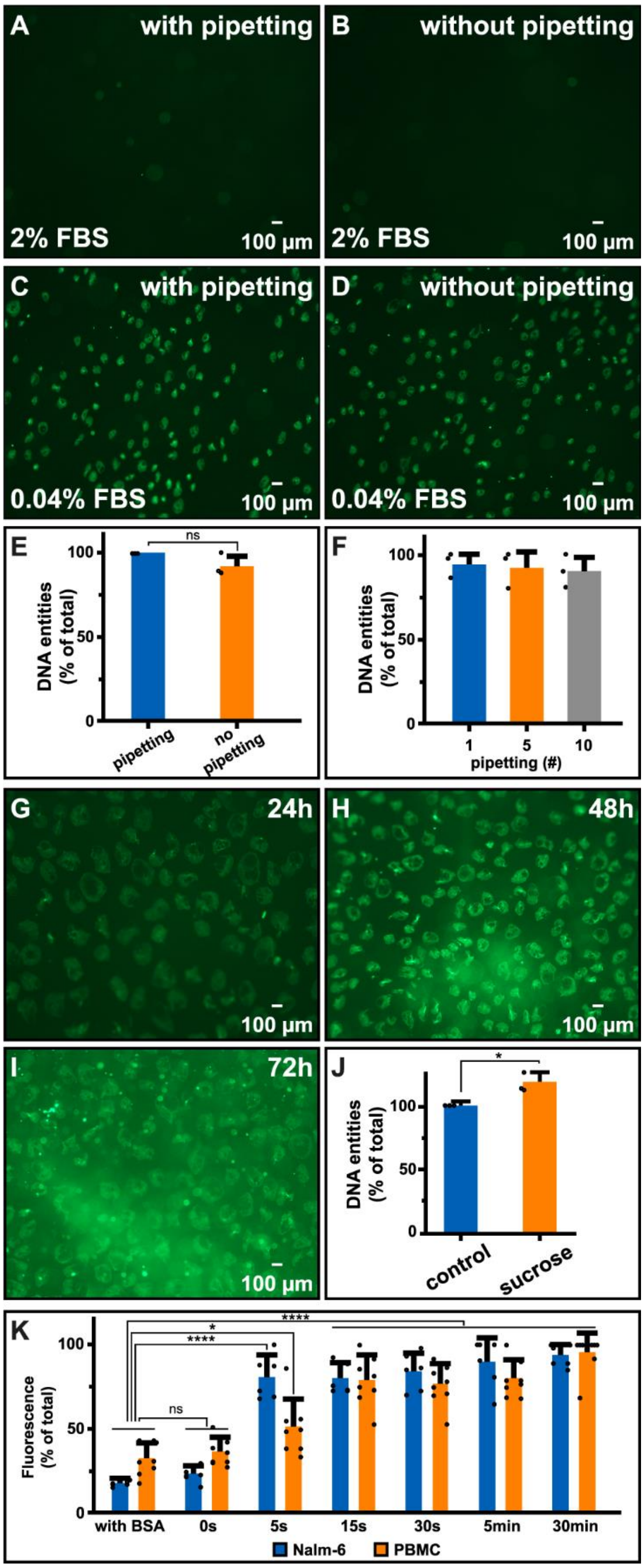


FIGURE. S2. **Extracellular DNA in serum-free media.** Visualization of extracellular DNA in supernatants released by Nalm-6 cells with and without resuspension by pipetting **(A-D)**. 300 000 Nalm-6 cells were resuspended in 10µl PBS containing 2% FBS and added to a 24 well plate to which 440 µl 2% FBS in PBS **(A and B)** or PBS **(C and D)** was added. Cells were either mixed by pipetting **(A and C)** or by gentle tilting of the plate **(B and D)**. **(E)** Relative quantification of released DNA entities shown in C and D. N=3. **(F)** The impact of pipetting on formation of extracellular DNA entities was examined by resuspend Nalm-6 cells in PBS using indicated number of pipetting events prior to seeding. No statistically significant differences were observed. N=3. Visualization of extracellular DNA in supernatants 24 **(G)** 48 **(H)** and 72 **(I)** hours after serum removal. **(J)**

Nalm-6 cells were seeded in serum-free RPMI-1640 with or without addition of 0.3 M sucrose to modify osmolality. Osmolality in the sucrose supplemented sample equals ~600 mOsm/kg while the control sample equals ~300 mOsm/kg. Three independent microscopy images for each sample were recorded and the number of DNA entities in these images were counted. The amount of DNA entities in control sample was considered 100%. The mean for each individual experiment (N=3) is shown (filled circle) and error bars represent standard deviation calculated using all obtained technical replicas. **(K)** Dedicated assessment of the timeframe for DNA release upon serum removal. Nalm-6 cells (N=6) and PBMC (N=8) were incubated in PBS for indicated times before BSA to a final concentration of 1mg/ml was added. SYTOX Green fluorescence in each sample was measured in a microplate reader. The sample with the highest fluorescence in each experiment was considered 100% to which all samples were compared. Filled circles represent % of max fluorescence in each experiment. Statistical analyzes were done using unpaired t test (E, J) or one-way ANOVA followed by Tukey's post-hoc test (F) or Dunnett's multiple comparisons test (K). * $P < 0.05$, **** $P<0.0001$.

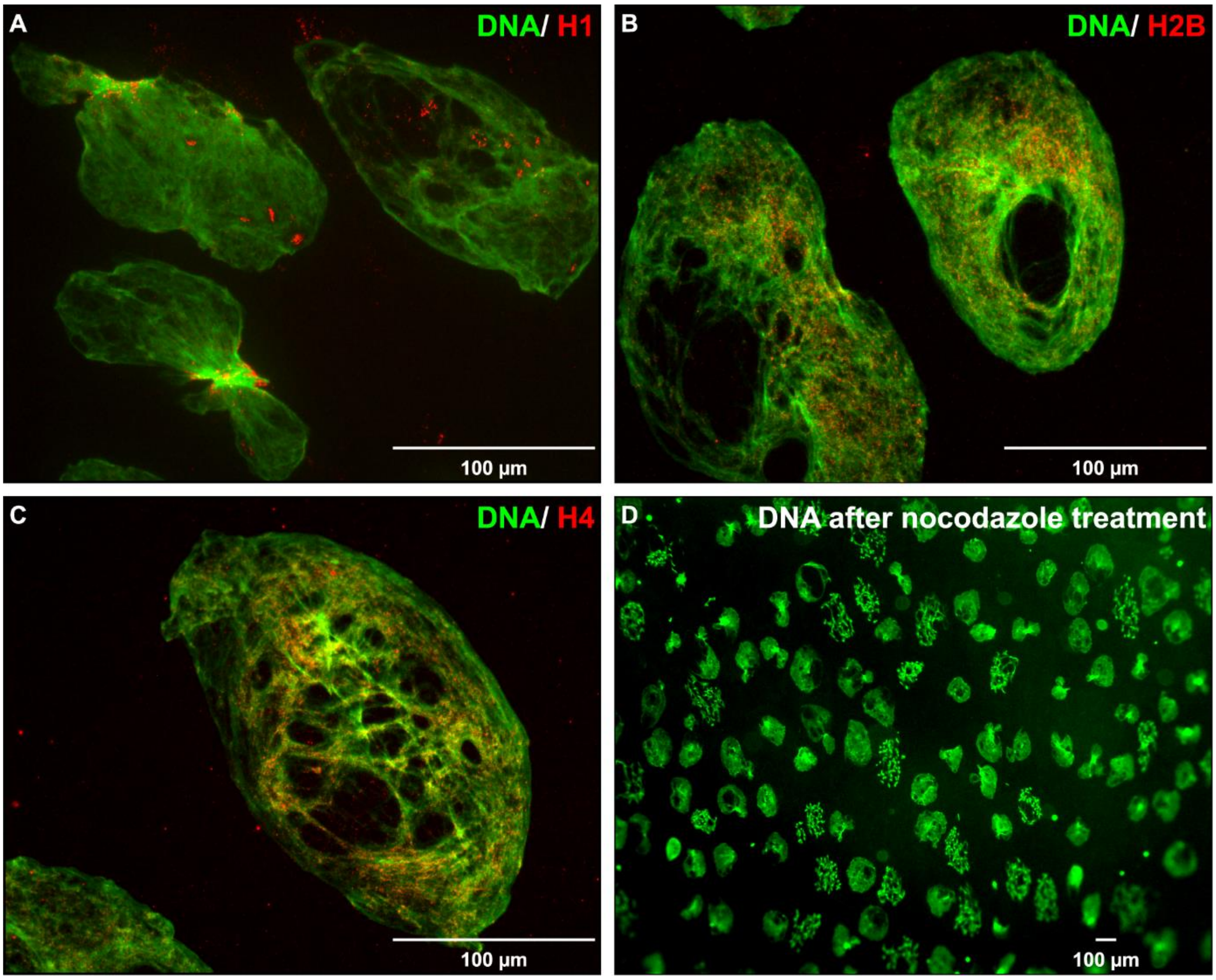


FIGURE S3. **Staining of DNA along with cellular components.** Microscopy images (40x objective) of extracellular DNA (green) released from Nalm-6 cells, immobilized on glass slides, and stained for different cellular components (red). **(A)** Immunostaining of Histone H1. **(B)** Immunostaining of Histone H2B. **(C)** Immunostaining of H4. **(D)** Microscopy image (4x objective) of extracellular DNA (green) in supernatant released from nocodazole treated Nalm-6 cells in serum-free medium.

**Table SI.** Antibodies used for immunolabeling of proteins prior to fluorescence microscopy.

| Antibody against | Product number | Supplier |
|---|---|---|
| **Centromere protein A** | GTX13939 | GeneTex |
| **Histone H1.2** | ab17677 | Abcam |
| **H2B** | 39123 | Active Motif |
| **H3** | | |
| C-term | 39163 | Active Motif |
| H3K9me2 | 39239 | Active Motif |
| **H4** | | |
| H4K8ac | AR-0138-200 | LP Bio |
| H4K16ac | 39167 | Active Motif |
| **Lamin B1** | SC-374015 AF488 | Santa Cruz Biotechnology |
| **SUN2** | SC-377459 | Santa Cruz Biotechnology |
| **Vimentin** | MS-129-P0 | NeoMarkers |

**Table SII.** Impact of polyethylene glycol (PEG) molecular size (Da) and concentration on DNA release. The amount of DNA entities in the PEG-free control sample to which all samples were compared was considered 100%. Results represent the mean of three independent experiments. Numbers within brackets represent standard deviations (n=3).

| | | **PEG molecular weight (Da)** | | | | | | |
|---|---|---|---|---|---|---|---|---|
| **conc.** | **control** | **200** | **400** | **600** | **1000** | **4000** | **6000** | **8000** |
| **2.5 nM** | 100 (2) | 103 (6) | 94 (10) | 91 (22) | 99 (2) | 105 (5) | 93 (6) | 74 (10) |
| **2.5 µM** | 100 (5) | 94 (10) | 84 (6) | 68 (6) | 20 (5) | 4 (1) | 5 (0) | 7 (1) |
| **2.5 mM** | 100 (6) | 75 (18) | 41 (14) | 29 (7) | 28 (7) | 5 (4) | 6 (4) | 8 (6) |
